# Improving Robotic Hand Prosthesis Control With Eye Tracking and Computer Vision: A Multimodal Approach Based on the Visuomotor Behavior of Grasping

**DOI:** 10.3389/frai.2021.744476

**Published:** 2022-01-25

**Authors:** Matteo Cognolato, Manfredo Atzori, Roger Gassert, Henning Müller

**Affiliations:** ^1^Institute of Information Systems, University of Applied Sciences and Arts of Western Switzerland (HES-SO Valais-Wallis), Sierre, Switzerland; ^2^Rehabilitation Engineering Laboratory, Department of Health Sciences and Technology, ETH Zurich, Zurich, Switzerland; ^3^Department of Neuroscience, University of Padua, Padua, Italy; ^4^Faculty of Medicine, University of Geneva, Geneva, Switzerland

**Keywords:** hand prosthetics, electromyography, deep learning, multi-modal machine learning, eye-tracking, eye-hand coordination, assistive robotics, manipulators

## Abstract

The complexity and dexterity of the human hand make the development of natural and robust control of hand prostheses challenging. Although a large number of control approaches were developed and investigated in the last decades, limited robustness in real-life conditions often prevented their application in clinical settings and in commercial products. In this paper, we investigate a multimodal approach that exploits the use of eye-hand coordination to improve the control of myoelectric hand prostheses. The analyzed data are from the publicly available MeganePro Dataset 1, that includes multimodal data from transradial amputees and able-bodied subjects while grasping numerous household objects with ten grasp types. A continuous grasp-type classification based on surface electromyography served as both intent detector and classifier. At the same time, the information provided by eye-hand coordination parameters, gaze data and object recognition in first-person videos allowed to identify the object a person aims to grasp. The results show that the inclusion of visual information significantly increases the average offline classification accuracy by up to 15.61 ± 4.22% for the transradial amputees and of up to 7.37 ± 3.52% for the able-bodied subjects, allowing trans-radial amputees to reach average classification accuracy comparable to intact subjects and suggesting that the robustness of hand prosthesis control based on grasp-type recognition can be significantly improved with the inclusion of visual information extracted by leveraging natural eye-hand coordination behavior and without placing additional cognitive burden on the user.

## 1. Introduction

The loss of a hand deprives an individual of an essential part of the body, and a prosthesis that can be controlled intuitively and reliably is therefore essential to effectively restore the missing functionality. Dexterous hand prostheses with notable mechanical capabilities are now commercially available. They commonly have independent digit actuation, active thumb opposition, sufficient grip force and sometimes a motorized wrist. These characteristics make such devices capable of performing a large variety of grasps that can substantially simplify the execution of activities of daily living (ADL) for hand amputees. On the other hand, to fully exploit these capabilities, the control system must be able to precisely and reliably decode the grasp the user intends to perform. Although numerous non-invasive strategies have been developed to achieve a robust and natural control for multifunction hand prostheses, their employment in commercial products and clinical practice is still limited (Castellini et al., 2014; Farina et al., 2014; Vujaklija et al., 2016). Pattern recognition-based approaches are arguably the most investigated ones in scientific research. They identify the grasp type by applying pattern recognition methods to the electrical activity of the remnant musculature recorded via surface electromyography (sEMG) (Hudgins et al., 1993; Scheme and Englehart, 2011; Jiang et al., 2012). Despite the remarkable performance obtained by these methods in controlled environments, they commonly have difficulty providing the level of robustness required for daily life activities in real-life conditions (Jiang et al., 2012; Castellini et al., 2014; Farina and Amsüss, 2016; Campbell et al., 2020). The intrinsic variability of the electromyographic signals and the presence of factors affecting them are arguably the main causes for the lack of robustness of pattern recognition-based myoelectric control methods (Farina et al., 2014; Campbell et al., 2020). Several strategies have been proposed to overcome these limitations, such as the development or selection of more robust signal features (e.g., Phinyomark et al., 2012; Khushaba et al., 2014, 2017; Al-Timemy et al., 2016), the application of more advanced methods of analysis, such as deep learning (e.g., Atzori et al., 2016; Geng et al., 2016; Faust et al., 2018), the use or addition of different modalities (e.g., Castellini et al., 2012; Gijsberts and Caputo, 2013; Jaquier et al., 2017), and the inclusion of complementary sources of information to increase the autonomy of the control system (e.g., Došen et al., 2010; Markovic et al., 2015; Amsuess et al., 2016). Despite all the mentioned difficulties, in the last years research achievements in pattern recognition helped to develop commercial pattern classification approaches, such as COAPT engineering ^1^ and Myo Plus from Ottobock ^2^. The idea of providing a prosthesis with decision-making capabilities is not novel (Tomovic and Boni, 1962), and several approaches have been proposed and investigated over the years. A promising method relies on identifying the most appropriate grasp type by obtaining information of the object a person aims to grasp (Došen and Popović, 2010; Markovic et al., 2014; Ghazaei et al., 2017; Taverne et al., 2019). In order to do so, the control system must have the ability to identify and extract information of the target object reliably, and the reliance on visual information is a common and natural strategy for this purpose. The use of visual modalities is motivated by the natural eye-hand coordination behavior humans use during grasping, where information to plan the motor action are retrieved by briefly fixating the object to be grasped before the hand movement (Land et al., 1999; Johansson et al., 2001; Land, 2006). Several studies have investigated the use of eye tracking techniques to improve the human-robot interaction during grasping. Castellini and colleagues have investigated the use of gaze to increase the level of autonomy of a robotic device in the context of teleoperation, imagining the possible benefit this approach could have in the control of prosthetic hands (Castellini and Sandini, 2007). In Corbett et al. (2012), an eye tracker was employed to improve the trajectory estimation of an assistive robotic hand for spinal cord injury patients. The authors concluded that the inclusion of gaze data not only improved the trajectory estimation but also reduced the burden placed on the user, facilitating the control. The electro-oculography technique was used in Hao et al. (2013) to extract object characteristics to pre-shape a hand prosthesis. In this work the participants were asked to scan the object's contour with their eyes, allowing the system to select the most suitable grasp type by predicting its affordances. Eye-hand coordination parameters were used in Cognolato et al. (2017) to semi-automatically extract patches of the object a person aims to grasp for the training of an object recognition system. Gigli et al. (2018) investigated the inclusion of information of the target object for grasp-type classification tasks. This evaluation exploited the use of sEMG as reaching phase detector, which triggers a fixation search to identify and segment the aimed object. Once the object is identified, a convolutional neural network (CNN) extracts visual features that are fused at kernel level with the sEMG modality. The results show a consistent improvement in classification accuracy for 5 able-bodied subjects with respect to the unimodal sEMG-based classification, showing that the inclusion of visual information produces an increment in grasp-type recognition robustness. The work by Gigli et al. (2018) sets a fundamental baseline in the domain of prosthetics. However, despite being on a similar topic and dataset, the previous work is strongly different from this paper. First, the authors did not include hand amputees in the dataset and they included a small number of intact subjects. Second, the approach was not fully based on deep neural networks. Third, it included the identification of fixations, which brings the disadvantage of shortening the time frame to identify the target object, that could prevent a correct identification (Gregori et al., 2019). Evaluations on transradial amputees are particularly desirable to better investigate the performance of multimodal approaches based on gaze and sEMG for prosthetic applications. This work aims at investigating the benefit of including visual information obtained by unobtrusively exploiting eye-hand coordination parameters in transradial amputees and able-bodied subjects to achieve an improved and more robust grasp-type classification for hand prosthesis control. To do so, we use the recently released MeganePro Dataset 1 (Cognolato et al., 2020), which includes sEMG, accelerometry, gaze, and first-person videos recorded from 15 transradial amputees and 30 able-bodied subjects while grasping several objects with ten grasp types. We used a Convolutional Long Short-Term Memory (ConvLSTM) network to perform sEMG-based grasp-type classification and a Mask Region-based Convolutional Neural Network (Mask R-CNN) (He et al., 2017) to identify and segment the objects in front of the subject. Eye-hand coordination parameters were used to identify the object the subject aims to grasp and the information from both modalities is combined for a final prediction.

## 2. Materials and Methods

### 2.1. Data

The data used in this work are publicly available in the MeganePro Dataset 1 (MDS1) (Cognolato et al., 2020). The sEMG data were collected at 1926 Hz using a Delsys Trigno Wireless EMG System (Delsys Inc., US). Gaze and first-person video were recorded at 100 Hz and 25 frames per second (FPS), respectively, with a Tobii Pro Glasses 2 eye tracker (Tobii AB, SE). The dataset contains recordings from 15 transradial amputees and 30 able-bodied subjects while grasping numerous household objects with ten grasp types. Twelve sEMG electrodes were placed around the forearm or residual limb in a two-array configuration. The acquisition protocol consisted of two parts. In the *static* condition, subjects were asked to statically perform 10 grasps, from a seated and a standing position. Each grasp was matched with three household objects chosen among a total of 18 objects as shown in Table 1) (Cognolato et al., 2020). Grasp-object pairings were chosen for having each grasp used with multiple objects and vice versa. At least five objects were placed in front of the subject, simulating a real environment. In the *dynamic* condition, the participants were asked to perform an action with the object after having grabbed it (i.e., opening a door handle or drinking from a can). The *dynamic* condition was repeated eight times on a set of two objects, either standing or seated. Explanatory videos, showed at the beginning of each grasp-block, instructed the participants on how to perform the grasps, and vocal instructions guided them through the exercises. The multiple relationships between objects and grasp types, as well as the simultaneous presence of several objects in the scene in front of the subjects, made the acquisition protocol similar to an everyday life scenario, providing a set of data suitable to investigate a multimodal control strategies based on sEMG, gaze and visual information.

**Table 1:** Overview of the grasp types and objects for the condition of the exercise.

	**Grasp**	**Object**
1	Medium wrap	Bottle
		Can
		Door handle
2	Lateral	Mug
		Key
		Pencil case
3	Parallel extension	Plate
		Book
		Drawer
4	Tripod grasp	Bottle
		Mug
		Drawer
5	Power sphere	Ball
		Bulb
		Key
6	Precision disk	Jar
		Bulb
		Ball
7	prismatic pinch	clothespin
		key
		can
8	Index finger extension	Remote
		Knife
		Fork
9	Adducted thumb	Screwdriver
		Remote
		Wrench
10	Prismatic four finger	Knife
		Fork
		Wrench

### 2.2. Unimodal sEMG-Based Grasp-Type Classification

We employed a ConvLSTM to perform the grasp-type classification based on solely electromyographic signals (**Figure 2**). One of the advantages of this network architecture is the capability of exploiting both spatial and temporal relationships of the data (Shi et al., 2015). This characteristic can positively impact the performance in this type of applications, where the recognition of a grasp type from the muscular activity of the extrinsic muscles of the hand can be discriminated by taking into account which muscle activates (i.e., via its location) and the temporal pattern of such contractions. The ConvLSTM network (Shi et al., 2015) is based on the widely used Long Short-Term Memory (LSTM) (Hochreiter and Schmidhuber, 1997) model. However, it differs from the LSTM conventional structure for the convolutional operations performed in both input and internal state transformations, which provide the capability of handling spatiotemporal information (Shi et al., 2015). The network used in this work consists of a Convolutional Long Short-Term Memory layer with 128 filters and a kernel size of 1 by 3, followed by a dropout layer with a rate of 0.5, a flatten layer, two fully connected layers of 200 and 50 units, respectively, with Rectified Linear Unit (ReLU) as activation function with a dropout having 0.2 as dropout rate. A fully connected layer with 11 units (the number of classes) and softmax as activation function provides the output of the network. We used the categorical cross entropy loss function and Adam (Kingma and Ba, 2014) as optimizer with the default parameters. The network was implemented using the Keras functional API.

**Figure 1:**
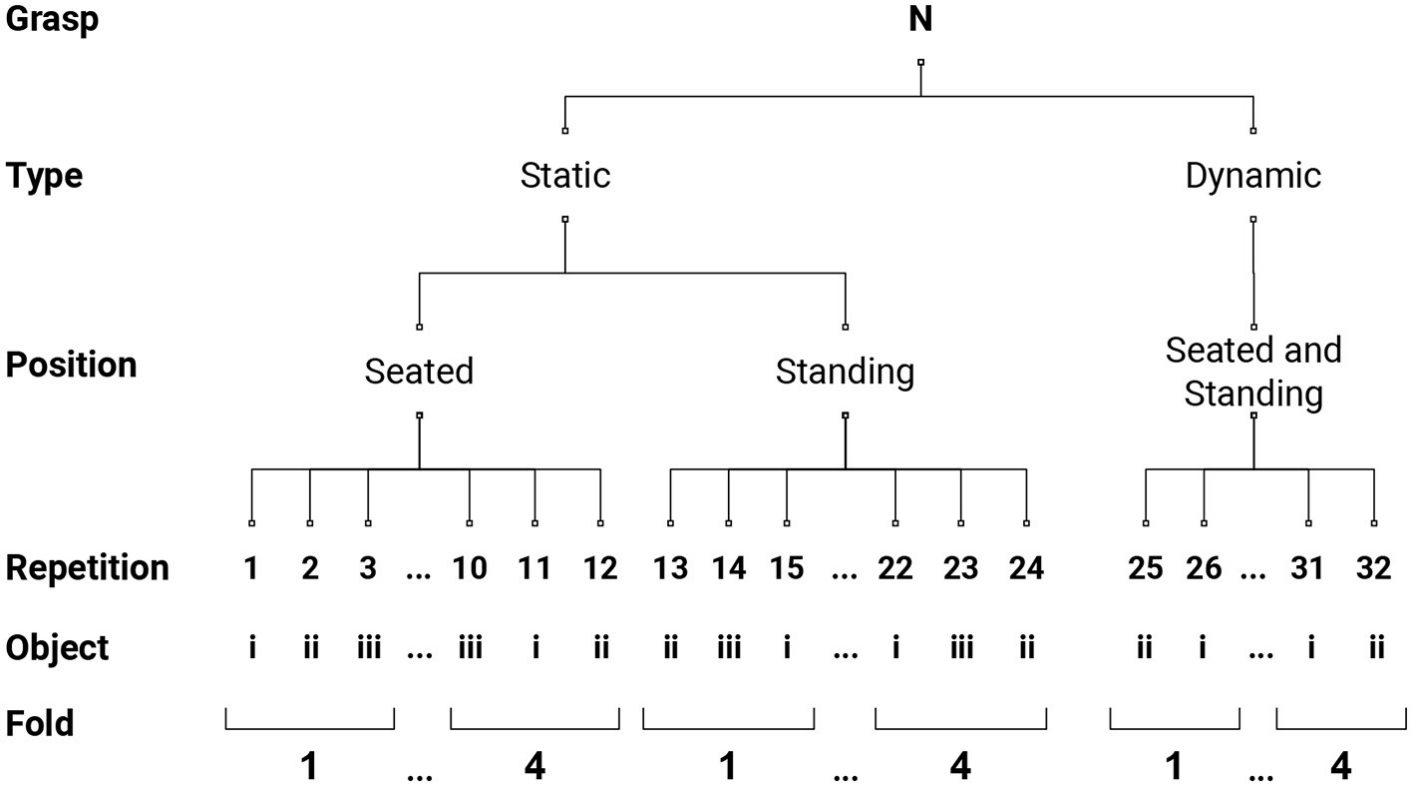
Four-fold data structure segmentation schema. As described in section 2.1, each grasp-type was performed on 2 conditions (*static* and *dynamic*) and repeated 32 times on different objects. Three repetitions from the *static* seated condition and from the *static* standing, and 2 from the *dynamic* condition formed a fold, which contains 8 repetitions per grasp-type.

The sEMG data were shaped following a rest-grasp-rest pattern, with a random amount of rest of a minimum of 1 s, and including part of the rest after the previous explanatory video for the first repetition. Therefore, a total of 320 sEMG segments were extracted per subject. We subsequently divided these segments into 4 folds, following the scheme employed in Cognolato et al. (2020) and graphically described in Figure 1. Each fold contains 8 repetitions per grasp-type, namely 3 repetitions from the *static* seated condition, 3 from the *static* standing, and 2 from the *dynamic*, performed on different objects. This allowed us to test the performance with a 4-fold cross-validation procedure, where 3 folds were used to train the network and the held-out folds for testing. For each training, a validation set of 30 repetitions was randomly drawn for each condition from the training set to evaluate the best model based on the validation accuracy. The validation set was obtained by randomly extracting a repetition from each condition per grasp type, resulting in a training set of 210 repetitions and a validation set of 30. We pre-processed all the data by removing the mean, making the variance unitary with a scaler that was fit on the training set and performing data rectification. After this, the data were windowed with a window size of 200 samples (slightly more than 100 ms) with no overlap, obtaining a *N* × 200 × 12 tensor, with N the number of windows and 12 the number of electrodes. Finally, each window was divided into 10 subsequences, each structured as a single row by 20 columns and 12 channels (one per electrode), obtaining an input tensor of shape *N* × 10 × 1 × 20 × 12 with which the ConvLSTM was fed. For each 4-fold cross-validation procedure, the network was trained for 150 epochs with batches of size 32, and the best models were saved based on the validation accuracy. The predominancy of the rest class was taken into account by providing the network with class weights.

### 2.3. Object Recognition and Segmentation

Object recognition and segmentation were performed with a Mask R-CNN network (He et al., 2017), utilizing the model released by Gregori et al. (2019). The model uses a ResNet-50-Feature Pyramid Network (He et al., 2016; Lin et al., 2017) as backbone. It is based on the implementation provided by Massa and Girshick (2018) that was originally trained on the Common Objects in Context (COCO) dataset (Lin et al., 2014) and fine-tuned on the MeganePro objects. Gregori et al. (2019) demonstrated the substantial increase in average precision of this model with respect to the non-fine-tuned model when tested on the on MeganePro objects, thanks also to the limited variability and number of objects employed in the MeganePro acquisitions. To reduce the computation time, we extracted and stored the contour of the objects identified by this network only from 2 s before to 3.5 s after the beginning of the grasp identified from the relabeled data. Furthermore, as done in Gregori et al. (2019), instances having a score level lower than 0.8 were discarded. At this stage, the gaze points within each video frame (at their original sampling rate) were used to identify the object being looked at by the subject. Similarly to what was done in Gregori et al. (2019), objects were considered looked at for grasping purposes when the gaze point was closer 20 px to the object contour (evaluated with the Euclidian distance). This is done only for *valid* gaze-frame instances that occur when the conditions of having a valid gaze-point estimation and at least one object recognized by the Mask R-CNN are both met (excluding the *person* and *background* “object” classes). The main advantages of this approach is the decoupling of object and grasp intention identifications, where the information about the object fixated by the user is instantly available once the grasp intention is detected (Gregori et al., 2019).

### 2.4. Multimodal Analysis

**Figure 2:**
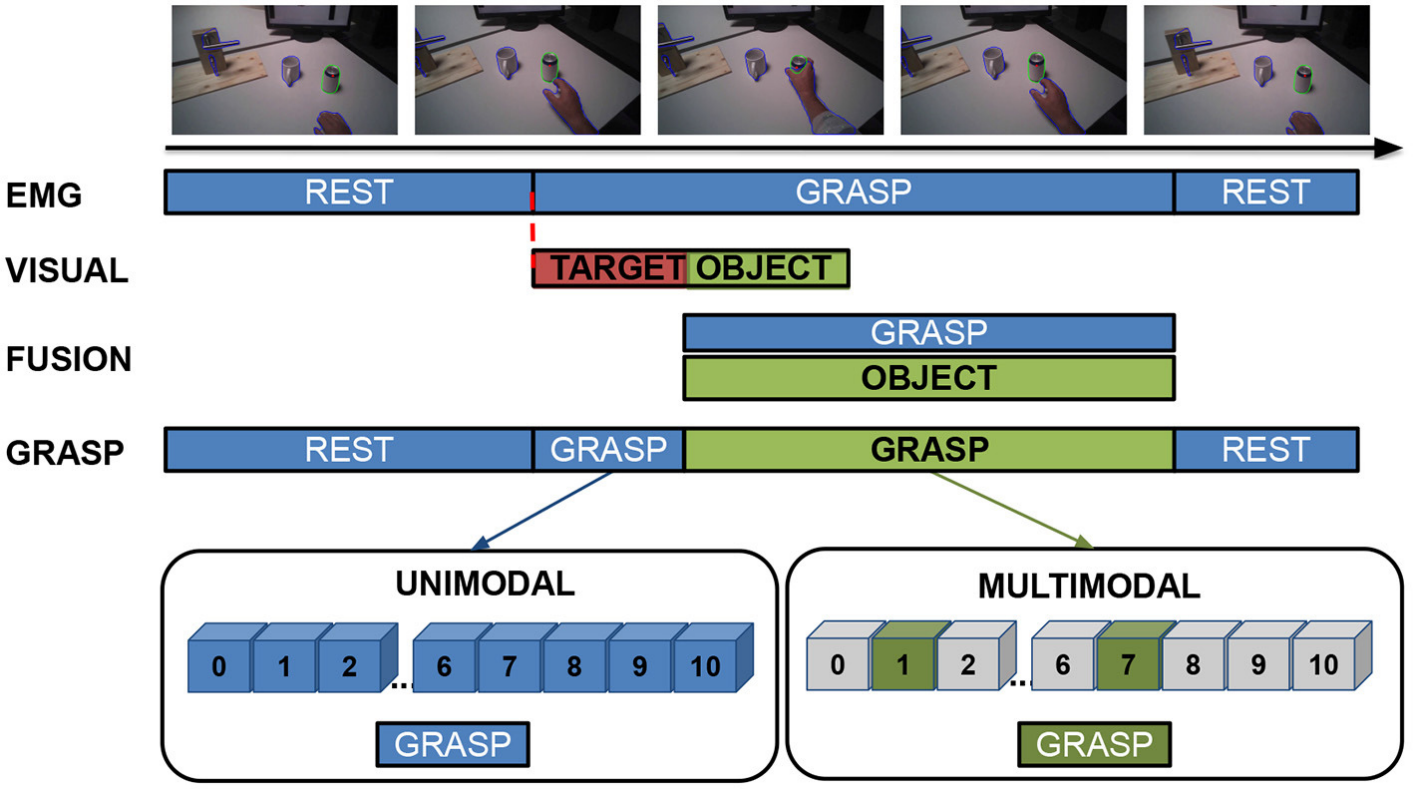
Example of a typical unimodal and multimodal analysis process flow. The sEMG-based grasp-type classification (*EMG* in the figure) continuously identifies the grasp type, and its output is taken as is when a *rest* condition or no object are detected (marked in blue). Once a grasp intent is identified, the offline-computed visual information are loaded and, if a target object is successfully identified, the final grasp is selected only among the grasp-types paired with the identified object (marked in green). The approach restarts as soon as a sample is classified as rest.

The multimodal analysis consists of fusing gaze, visual data and sEMG with the aim of increasing the robustness of grasp-type recognition (Figure 2). The multimodal analysis was performed on the data extracted with the Mask R-CNN, namely in the time frame of 2 s before and 3.5 s after the beginning of the grasp, maintaining the same 4-fold structure used for training the ConvLSTM models. The sEMG-based grasp-type classification was performed every 20 samples (approximately 10 ms) with the best models obtained in the unimodal sEMG-based grasp-type classification step (section 2.2). This served to both identify the beginning of a grasp and classify the grasp type based only on the sEMG. The identification of the grasp intent is obtained by leveraging the ability of the ConvLSTM to differentiate between rest and grasp, and the multimodal data fusion is triggered only after the recognition of a *non-rest* condition (i.e., when a grasp type is detected, regardless of its type). To perform the multimodal data fusion, the information stored during the object recognition and segmentation step (section 2.3) is loaded each time a new valid gaze-frame instance is available. Once a grasp intention is detected, the target object is then identified as the last object being looked at in the previous 480 samples (approximately 250 ms). If no object is identified, the search continues until 500 ms after the grasp intention identification. In the case that an object is successfully identified, the grasp types paired with the recognized object are fused with the information provided by the sEMG-based classifier. In particular, the final grasp type is chosen as the one having the highest rate in the output vector of the ConvLSTM restricted to the grasp types paired with the recognized object. If no object is identified within this time-frame, the approach continues with only the sEMG-based grasp-type classification. In both cases (i.e., whether an object is identified or not), the approach restarts as soon as a sample is classified as rest. In the subsequent analysis, the subject identified as *S114* was excluded from the multimodal analysis due to the strabism condition that negatively influenced the quality of the eye tracking data (Cognolato et al., 2020).

### 2.5. Statistical Analysis

Two non-parametric statistical tests were applied to evaluate the significance of the results: the Wilcoxon signed-rank test for paired samples and the Mann-Whitney test for independent samples. We applied the Wilcoxon signed-rank test to results obtained from the same population group (i.e., amputees or non-disabled subjects). This test validates the variations of using different approaches (e.g., sEMG-based vs. multimodal) or conditions (e.g., static vs. dynamic) on the same population data. The Mann-Whitney test was used to evaluate if the discrepancy in correct object identification between the two population groups (e.g., amputees vs. able-bodied subjects) was statistically significant. Non-parametric tests were chosen to cope with the non-normal data distribution. When comparing the sEMG-based and multimodal approaches with the Wilcoxon signed-rank test, we used the alternative hypothesis that the multimodal approach performs better than the unimodal. A null hypothesis of a difference between the conditions was used for the correct object identification comparison. The matched rank biserial correlation and the rank biserial correlation provided the effect size for the Wilcoxon signed-rank test and the Mann-Whitney test, respectively, and the values reported hereafter represent the magnitude of the effect size. The statistical analysis was applied to the results averaged per subject and was performed with JASP (JASP Team, 2020).

## 3. Results

The results show that exploiting eye-hand coordination (via the fusion of electromyography, gaze, and first-person video data) significantly increases the average classification accuracy for both intact subjects and amputees, suggesting that the robustness of hand prosthesis control based on grasp-type recognition can be improved significantly with the inclusion of visual information.

The next sections present the results obtained with the unimodal sEMG-based grasp-type classification, the rate of correct target object identification and the performance achieved with the multimodal approach. The accuracy was evaluated per fold, while the rate of correct object identification is evaluated per subject.

### 3.1. Unimodal sEMG-Based Grasp-Type Classification

The first step consisted in evaluating the performance of the ConvLSTM on sEMG-based grasp-type classification. The average classification accuracies obtained with the 4-fold cross validation approach presented in section 2.2 on the 11 classes (ten grasp types and rest) are of 72.51 ± 5.31% and 74.54 ± 4.96% for transradial amputees and able-bodied subjects, respectively.

### 3.2. Object Recognition via Eye-Hand Coordination Parameters

**Figure 3:**
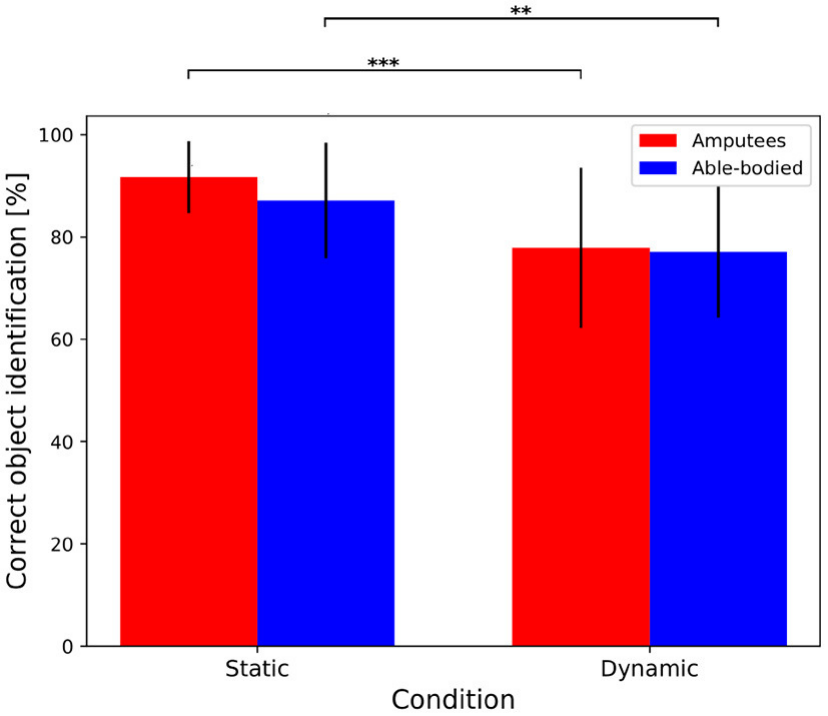
Object recognition rate via eye-hand coordination parameters for transradial amputees and able-bodied subjects in both *static* and *dynamic* conditions. The bar illustrates the mean value and the error bars the standard deviation. **p < 0.01, and ***p < 0.001.

This section aims at evaluating whether the target object was correctly identified by using eye-hand coordination parameters. This is crucial to assess the actual performance of the approach, since the correct grasp type may still be retrieved even with an incorrect identification of the target object, given that multiple objects graspable with the same grasp type are always present in the scene. Therefore, this protocol-related aspect might introduce a bias that can boost the results. To evaluate this aspect, we considered an identification as correct when the object recognized by the multimodal approach at the beginning of the grasp corresponds to the target one. The average rate of correct object identification is higher than 85 % for both transradial amputees (91.88 ± 6.80%) and able-bodied subjects (86.08 ± 11.00%) for the *static* condition, indicating that the correct object was recognized for the vast majority of the trials. These values slightly decrease for the *dynamic* condition, where the correct object was identified for the 79.73 ± 15.54% of the times for transradial amputees, and for the 79.50 ± 12.14% for able-bodied subjects. The statistical analysis revealed that the condition (*static* or *dynamic*) has a statistically significant effect on the correct object recognition rate (*p* < 0.001 and *p* = 0.001 for amputees and able-bodied subjects, respectively), while no significant difference is found between the groups (*p* = 0.051 and 0.441 for the *static* and *dynamic* conditions, respectively). Figure 3 shows the results for both groups and conditions while the outcomes of the statistical analysis are summarized in Table 2.

**Table 2:** Within-subject (left) and between-subjects (right) statistical description of the correct object recognition rate.

	**Amputees**		**Able-bodied**			***Static***		**Functional**	
	***p*** **-value**	**Effect size**	***p*** **-value**	**Effect size**		***p*** **-value**	**Effect size**	***P*** **-value**	**Effect size**
Static - Dynamic	<0.001	0.943	0.001	0.699	Amputees - Able-bodied	0.051	0.371	0.441	0.148

*The table on the left reports the statistical analysis performed with the Wilcoxon test to evaluate the effect of the static and dynamic conditions within the same population group. The evaluation of differences among amputees and able-bodied subjects for the two conditions obtained with the Mann-Whitney test is reported on the right*.

### 3.3. Multimodal Analysis

This section reports the results obtained by applying the multimodal approach presented in section 2.4. To better investigate the possible condition-related differences, the results are reported for the *static* and *dynamic* condition separately. It is worth noticing that, in this section, the unimodal and multimodal approaches are evaluated on the same data (segmented as described in section 2.4), as, due to differences in data segmentation, the results of the multimodal analysis are not directly comparable to the ones described in the unimodal sEMG-based grasp-type classification section (section 3.1). In fact, while the unimodal approach previously presented was tested on entire repetitions, in this section the testing was performed on the data extracted in the object recognition and segmentation phase, namely from 2 s before to 3.5 s after the beginning of the grasp identified from the relabeled data.

#### 3.3.1. Static Condition

**Figure 4:**
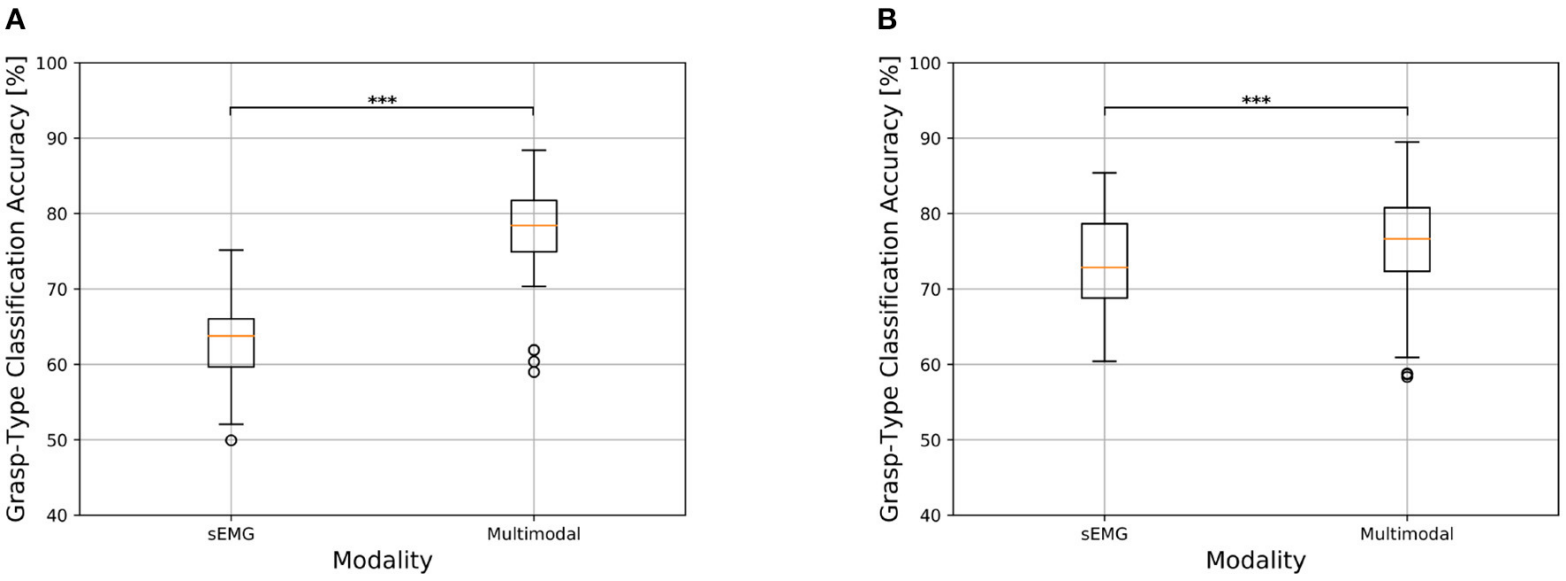
Comparison of grasp-type classification accuracy obtained with sEMG-based and multimodal approaches in transradial amputees and able-bodied subjects for the *static* condition. The top and bottom of the box indicate the third and first quartiles, respectively. The central line reports the median, the whisker extension follows the original definition. Data falling outside 1.5 times the interquartile range are considered outliers and indicated with circles. *p < 0.05, **p < 0.01, and ***p < 0.001. **(A)** Grasp-type classification in transradial amputees for the *static* condition. **(B)** Grasp-type classification in able-bodied subjects for the *static* condition.

The inclusion of gaze and visual information has led to a substantial increase in grasp-type classification accuracy for both the amputee and able-bodied groups for the static condition. For amputees in particular, the average classification accuracy obtained using only the sEMG modality was of 63.03 ± 5.36% while the multimodal approach reaches on average 78.64 ± 6.13%, with an average increment of 15.61 ± 4.22% (Figure 4A). This difference is found to be statistically significant (*p* < 0.001, effect size = 1, Figure 4B), indicating that the multimodal approach significantly increases the grasp-type classification accuracy. The same trend, even though to a reduced extent, was obtained for able-bodied subjects that achieve an average increment of 6.56 ± 2.89%, where the approach based only on sEMG data reached an average accuracy of 73.35 ± 6.14% while the multimodal analysis 79.92 ± 5.85% (Table 3). Also in this case, the increase in classification accuracy with the multimodal approach was found to be statistically significant (*p* < 0.001, effect size = 0.996, Table 3). The confusion matrices for the grasp-type classification of the *static* condition are reported in the Supplementary Material section.

**Table 3:** Statistical analysis of the accuracy achieved with the unimodal and multimodal approaches for the *static* and *dynamic* conditions.

	**Amputees**		**Able-bodied**	
	***p*-value**	**Effect size**	***p*-value**	**Effect size**
Static	<0.001	1.000	<0.001	0.996
Dynamic	<0.001	1.000	<0.001	0.991

*The analysis was performed with the Wilcoxon test with the alternative assumption that the results obtained with the unimodal approach were lower than the multimodal ones*.

#### 3.3.2. Dynamic Condition

**Figure 5:**
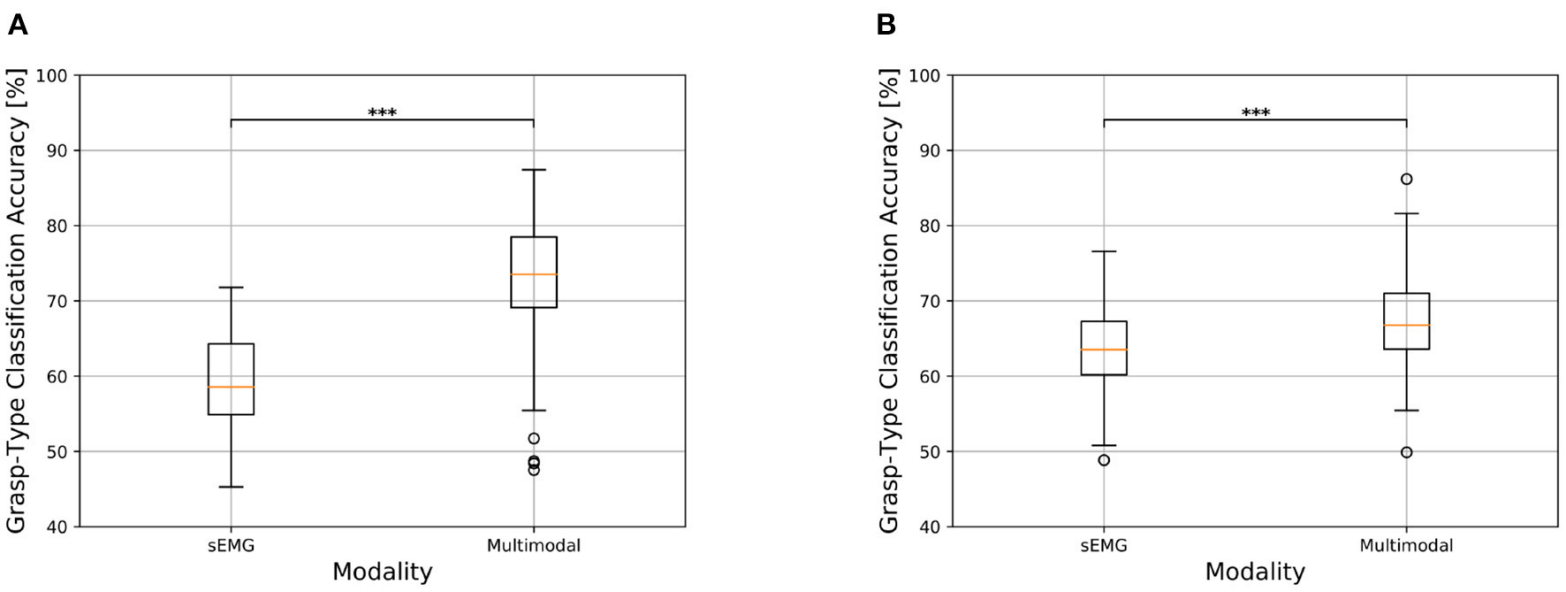
Comparison of grasp-type classification accuracy obtained with sEMG-based and multimodal approaches in transradial amputees and able-bodied subjects for the *dynamic* condition. See Figure 4 for information about boxplots' parameters. *p < 0.05, **p < 0.01, and ***p < 0.001. **(A)** Transradial amputees. **(B)** Able-bodied subjects.

The trend obtained for the *static* condition is also maintained for the *dynamic* one, where there is however an overall decrease in classification accuracy. Hand amputees reached 58.99 ± 6.26% and 74.12 ± 8.87% as average grasp-type classification accuracy for the sEMG-based and multimodal methods, respectively, with an average increment of 15.13 ± 6.32% (Figure 5A). The Wilcoxon test revealed the statistical significance of these results (*p* < 0.001, effect size = 1, Table 3), which indicates that the multimodal approach leads to a significant increase in grasp-type classification accuracy in hand amputees also for the *dynamic* condition. Focusing on the able-bodied subjects, the inclusion of visual information led to an average increase of 7.37 ± 3.52%, with the sEMG-based grasp-type recognition and the multimodal approach reaching an average accuracy of 63.55 ± 5.23% and 70.92 ± 5.40%, respectively (Figure 5B). Also in this case, the increase in performance obtained with the multimodal approach is statistically significant (*p* < 0.001, effect size = 0.991, Table 3). The confusion matrices for the grasp-type classification of the *dynamic* condition are reported in the Supplementary Material section).

## 4. Discussion

This work shows that a multimodal approach based on eye-hand coordination (thus fusing sEMG, gaze, and visual data) can significantly improve the performance of a grasp-type classifier, particularly for trans-radial amputees, suggesting that the robustness of hand prosthesis control can be improved with the inclusion of visual information. Furthermore, the results indicate that the target object can be recognized with a high probability by relying on the eye-hand coordination parameters without placing any additional burden on the user.

Target object recognition is overall slightly higher in amputees, without significant difference between the two populations (Table 2, *p* = 0.051 and 0.441 for the *static* and *dynamic* conditions, respectively). This result suggests that an accurate identification of the target object can be accomplished: (1) by leveraging the ability of the classifier to discriminate between rest and grasp, serving as an intention detector, (2) by exploiting the timing of visuomotor coordination, and (3) by continuously tracking the objects that the user is looking at (as proposed in Gregori et al., 2019). The use of the classifier as both intent detector and grasp-type classifier eliminates the need for a two-step approach in which the grasp-type classification is performed *after* the identification of the movement onset, which can reduce the time window to identify the target object correctly. However, the use of an sEMG-based classifier as an intention detector still does not allow to exploit the entire time window for the identification of the target object, as a latency between the start of a movement and the muscle activation exists and was shown to be significantly longer for amputees (Gregori et al., 2019). The inclusion of forearm kinematics [e.g., via via inertial measurement units (IMUs)] can probably improve this aspect, even though a muscular activation clearly marks the user intention of moving the hand, while arm kinematics intrinsically have a higher degree of variability, particularly in unconstrained environments where the rest-grasp transition might not be clearly identifiable. Furthermore, the use of a continuous gaze and object tracking to identify the object looked at by the user eliminated also the need to recognize the gaze fixation on the target object, which is not a trivial task and it has the disadvantage of shortening the time window for the grasp identification. The main disadvantage of a continuous gaze and object tracking, however, is the computational demand.

On the other hand, tools such as Mask R-CNN and You Only Look Once (YOLO) (Redmon and Farhadi, 2018) can perform object detection and segmentation in real-time, which can be made more efficient by restricting the detection area to the surroundings of the gaze point.

The results show a gap of approximately 10 % in the correct object identification between the *static* and *dynamic* conditions, which was found to be statistically significant. This difference may be due to the tasks that are performed under the two conditions. First, the increased number of objects placed in the scene for the *dynamic* condition and therefore their spatial vicinity can facilitate the selection of an object near the target one, resulting in incorrect identification. Second, the different gaze behavior shown in Gregori et al. (2019) for *in-place, lifting*, and *displacement* actions could also have influenced the correct object identification. For *in-place* actions (i.e., when the object is not moved), the participants only had to localize the target object in order to plan the motor action accordingly (i.e., defined as *locating* fixation by Land et al., 1999) (Land et al., 1999; Johansson et al., 2001; Land, 2006; Gregori et al., 2019). Instead of this, a series of activities were requested for the *lifting* and *displacement* actions (contained only in the *dynamic* condition), which commonly cause a gaze shift before the current action is completed, in order to plan the next step (Land et al., 1999; Johansson et al., 2001; Land, 2006; Gregori et al., 2019). Finally, no significant difference was found between the amputees and able-bodied subjects within each condition for what concerns the target object identification. This is consistent with the results from Gregori et al. (2019), where similar visuomotor strategies were found for the two groups.

A comparison of the results for the unimodal sEMG-based grasp-type classification with the state of the art is not easy to perform due to the differences in data, protocols, and subjects. Although different for data segmentation, the closest investigation in terms of data and protocol is the validation given in Cognolato et al. (2020). The grasp-type classification accuracies achieved in this work with the ConvLSTM are in line with those in Cognolato et al. (2020). Although the Kernel Regularized Least Squares with a nonlinear exponential χ^2^ kernel and marginal Discrete Wavelet Transform features achieved better performance (Cognolato et al., 2020), the main advantages of using ConvLSTM are the complete absence of the feature extraction step, and the use of shorter time-windows. The difference in unimodal sEMG-based grasp-type classification accuracy between amputees and intact subjects is roughly 2 %. This result is in line with the findings from Cognolato et al. (2020), where traditional and well-established machine learning approaches were employed. Another point that merits further analysis is the extent to which the displacement of a real object for the able-bodied subject (which was not “materially” performed by the amputees) might have influenced the comparison between the two groups, as it is well known that changes in force level negatively influence the sEMG-based grasp-type classification (Campbell et al., 2020).

The inclusion of visual information substantially increased the average grasp-type classification accuracy. This trend is maintained for both the *static* and *dynamic* conditions, with an average accuracy gain of approximately 15 % for hand amputees and roughly 7 % for intact subjects, and it is found to be statistically significant for both population groups. These results reveal the benefit of merging gaze and visual information with the traditional sEMG-based approach to improve the grasp type classification, and it is in line with the findings of Gigli et al. (2018) and Gregori (2019). Although the different data segmentation makes the results not directly comparable, our results showed a stronger increase for both populations than the one reported in Gigli et al. (2018) and Gregori (2019). In addition to the different data segmentation, also the different methods for performing the sEMG-based grasp-type recognition, for extracting the visual information and for performing the data fusion have likely contributed to this difference. On the other hand, both approaches indicate the benefit of including visual information for grasp-type recognition for both populations, with the amputees showing an increase roughly twice the one of the able-bodied subjects. Further efforts should be put to analyze the reasons behind the different increase in classification accuracy between the two populations. A possible hypothesis, to be verified in future works, is that the different increase of classification accuracy between the two populations might be due to the fact that the performance of the computer vision part of the pipeline (which is similar in the two groups) is capable to fully counterbalance the low performance of sEMG until a certain level of accuracy.

It should be noted that the approach proposed in this work performs a grasp-type classification based on the sEMG modality and the information about the suitable grasp types for the target object is merged *after* the classification step. In addition, this information is used to select the class with the highest recognition rate among those paired with the target object. Therefore, it seems reasonable that this approach has more influence in cases of uncertainty between classes (i.e., when several classes are similarly likely to be correct) than when the classifier assigns a high probability to one of the classes (either correct or not) paired to the object. The approach can exclude grasp types with a similar likelihood to the correct one in the former scenario, thereby improving the recognition of the appropriate class, whereas it has no effect if a high probability is given to an incorrect class among the suitable ones. It is also worth noticing that both performance and improvements depend on the chosen classifier as well as on its ability to correctly identify a grasp type, as it seems reasonable that the benefit of including additional and complementary sources of information decreases as the performance of the unimodal classification increases. However, given that a greater improvement for amputees was also obtained in Gregori (2019) with a different approach, further investigations might enlighten the reason of this difference.

The proposed method indicates the viability of taking advantage of a natural human behavior to improve the grasp-type recognition by having the control system retrieving complementary information autonomously, without placing any additional burden on the user. Furthermore, the approach is mainly driven by the sEMG modality, which is the direct user-device interface, making the autonomous part of the method as unobtrusive as possible. This was done in an effort to limit the conscious and visual attention demands, which was deemed as an aspect needing improvements by myoelectric prosthesis users (Atkins et al., 1996; Cordella et al., 2016).

Having the data analysis procedure fully based on deep neural networks can lead to the seamless integration of different modalities and to faster models, particularly at the testing phase (e.g., Ren et al., 2015). The first point represents a potential starting point for future work targeting multimodal data analysis employing multiple data acquisition techniques. The second one can lead to better real-time applications, obtained by reducing the number of separate processes that are required to achieve the same task.

Finally, the multimodal approach showed a small increment in misclassification toward the rest class. This increase of misclassifications might be a consequence of the “releasing” strategy employed in the approach, where a rest sample from the unimodal sEMG-based grasp-type classification marks the end of the prehension. In this case, the control returns to be purely sEMG-based, re-initializing the search for a new target object, which is improbable to be found for the *dynamic* condition, as the gaze has likely been moved to the next activity “step.” A more robust identification of the prehension completion, for example by requiring a minimum number of consecutive rest samples instead of a single one, could improve this aspect. On the other hand, this would increase the delay between the user intent and prosthesis reaction, reducing the speed of the control in an online application.

### 4.1. Limitations and Further Improvements

In order to achieve our objectives, we decided to limit additional uncertainties regarding the object recognition and the grasp-types paired with it. However, in a real scenario, the object recognition accuracy is likely to be lower than the one achieved in this work with a network fine-tuned on the objects composing the acquisition setup (Gregori et al., 2019), and the grasp types suitable for a specific object are commonly not fully known a priori. Both aspects can influence the improvement achievable.

A limitation of this work is that the computer vision pipeline was tuned on the same objects that were used in the acquisition protocol. This approach was applied to be consistent with the electromyography data analysis procedure. Nevertheless, object recognition accuracy in a real scenario is likely to be lower than the one achieved in our work. A perspective of object recognition for grasping without fine tuning can be found in Gigli et al. (2018), showing that multimodal fusion increases the classification success rate considerably, even if fine tuning is not performed. An alternative perspective of object recognition for grasping by exploiting dedicated training datasets in provided by Ghazaei et al. (2017). Despite the fact that in a real scenario object recognition accuracy is likely to be lower than the one achieved in our work, we expect this aspect to be improved in the future thanks to new resources which are being developed, increasing the applicability to real life applications. In fact, the presented work is one of the first approaches exploring what can be done fusing electromyography, computer vision and eye tracking data using deep learning approaches to mimic human eye-hand coordination. In this moment, real-life applications would at least benefit from fine tuning models on dedicated datasets (Ghazaei et al., 2017), bringing their performance at least closer to the ones described in this paper, and of newer computer vision architectures. In addition, real life applications of this system in products will most likely require years, during which performance in computer vision will probably continue to advance, with dedicated architectures, leading to better models for grasp classification too, even without or with limited fine-tuning.” On the other hand, information on the suitable grasp types can be extracted from the first-person video without the need of recognizing the object, for example by evaluating the object's characteristics (e.g., shape, size) with computer vision approaches (Došen et al., 2010; Hao et al., 2013; Markovic et al., 2015), deep learning techniques (Redmon and Angelova, 2015; Ghazaei et al., 2017; Gigli et al., 2018; Taverne et al., 2019), or by evaluating its affordances (Nguyen et al., 2016). Moreover, the position of the gaze on the object can also help to discriminate among multiple affordances, as objects can commonly be grabbed with several grasp types (e.g., if the gaze point is on the bottle cap, it is more likely that the user is planning to open the bottle, thus suggesting the use of a *tripod* grasp). Considering the unimodal sEMG-based grasp-type classification, although the chosen network achieved results in line with the one shown for the dataset validation (Cognolato et al., 2020), other networks and architectures might further improve the performance, which may also limit the potential benefit of including complementary information.

An additional point concerns the data fusion, because when the approach fails to detect the correct object an incorrect grasp type is likely to be chosen. A further refinement may select the final grasp type by taking into account the confidence of the recognition from both modalities, weighting the final selection toward the most promising one. Considering that rest is equally classified in unimodal and multimodal analysis, fully including it might influence the classification performance, possibly reducing the difference in performance between the two approaches. The need to wear an eye tracker might affect the usability of the setup. On the other hand, novel devices similar to normal eyeglasses are now on the market and it is plausible to think of future improvements that can make this technology even less obtrusive, for example by integrating it into standard glasses or even in contact lenses (Sako et al., 2016; Pupil Labs, 2020).

Finally, to validate the viability and performance of the approach, it should be implemented and tested in a real-time fashion with transradial amputees, possibly during the execution of ADL in unconstrained environments.

## 5. Conclusion

The aim of this work was to investigate if a multimodal approach leveraging a natural human behavior (i.e., the eye–hand coordination) can improve the challenge of classifying several grasp types for hand prosthesis control. The results are encouraging, showing that the fusion of electromyography, gaze, and first-person video data increases the offline grasp-type classification performance of transradial amputees. We used the publicly available MeganePro Dataset 1, containing sEMG, gaze, first-person video data collected from 15 transradial amputees and 30 able-bodied subjects performing grasping tasks on household objects in *static* and *dynamic* conditions. A deep neural network architecture based on a ConvLSTM performs the unimodal sEMG-based grasp-type classification, while the object recognition and segmentation are executed with a Mask R-CNN. A grasp-type classification based on the sEMG is continuously performed, allowing to identify grasp intents by leveraging the ability of the network to distinguish between the resting and grasping conditions. The identification of a grasp intent triggers the search for the target object based on eye-hand coordination parameters and in the case of an object being identified, the grasp type is selected among the suitable ones for the recognized objects. Otherwise, the approach continues the grasp-type classification relying only on the sEMG modality. The results show that the multimodal approach significantly increases the performance in transradial amputees and able-bodied subjects. In both the *static* and *dynamic* conditions, the performance increment obtained with the multimodal approach allowed the grasp-type classification accuracy in transradial amputees to be comparable with the one obtained in able-bodied subjects, without placing additional control burden on the user. The results therefore show the benefit of a multimodal grasp-type classification and suggest the usefulness of the approach based on eye-hand coordination. Moreover, the availability of the dataset allows for further investigations and improvements, which are desirable to obtain an approach that can be tested in online applications.

## Data Availability Statement

The datasets presented in this study can be found in online repositories. The names of the repository/repositories and accession number(s) can be found here: doi: 10.7910/DVN/1Z3IOM.

## Ethics Statement

The experiment was designed and conducted in accordance with the principles expressed in the Declaration of Helsinki. Ethical approval for our study was requested to and approved by the Ethics Commission of the canton of Valais in Switzerland (CCVEM 010/11) and by the Ethics Commission of the Province of Padova in Italy (NRC AOP1010, CESC 4078/AO/17). Prior to the experiment, each subject was given a detailed written and oral explanation of the experimental setup and protocol. They were then required to give informed consent to participate in the research study.

## Author Contributions

MC contributed to the design of the multimodal approach, performed the data analysis, and wrote the manuscript. MA contributed to the design of the multimodal approach, to the ideation of the data analysis procedure, and revised the manuscript. RG contributed to the design of the multimodal approach and revised the manuscript. HM contributed to the design of the multimodal approach, to the conception of the data analysis procedure, and revised the manuscript. All authors contributed to manuscript revision, read and approved the submitted version.

## Funding

This work was partially supported by the Swiss National Science Foundation Sinergia project #160837 MeganePro.

## Conflict of Interest

The authors declare that the research was conducted in the absence of any commercial or financial relationships that could be construed as a potential conflict of interest.

## Publisher's Note

All claims expressed in this article are solely those of the authors and do not necessarily represent those of their affiliated organizations, or those of the publisher, the editors and the reviewers. Any product that may be evaluated in this article, or claim that may be made by its manufacturer, is not guaranteed or endorsed by the publisher.

## Footnotes

^1^ http://www.coaptengineering.com/

^2^ https://www.ottobockus.com/prosthetics/upper-limb-prosthetics/solution-overview/myo-plus/myo-plus.html
