# Supplementary material for: Improving Robotic Hand Prosthesis Control With Eye Tracking and Computer Vision: A Multimodal Approach Based on the Visuomotor Behavior of Grasping
Source: Front Artif Intell. 2022 Jan 25;4:744476. doi: 10.3389/frai.2021.744476 (PMC8822121; doi:10.3389/frai.2021.744476)
Supplement: Supplementary file 1 [file Data_Sheet_1.pdf]

Supplementary Material

1 SUPPLEMENTARY DATA

This section reports the confusion matrices for the two populations under the static and functional conditions.

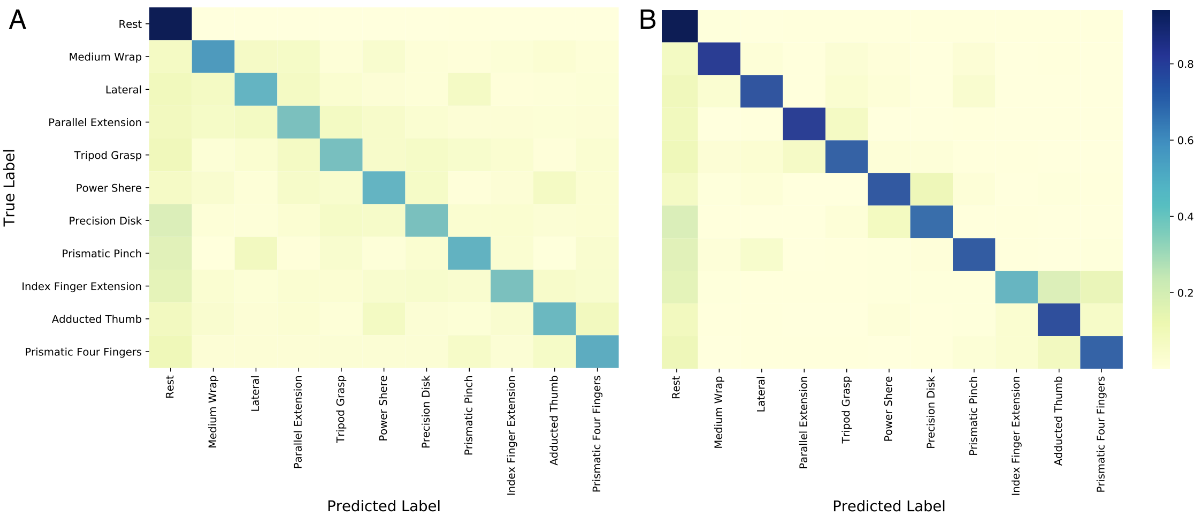

Figure S1. Normalized confusion matrices of sEMG-based and multimodal grasp-type classifications in transradial amputees subjects for the static condition. **(A)** Confusion matrix of sEMG-based grasp-type classification. **(B)** Confusion matrix of multimodal grasp-type classification.

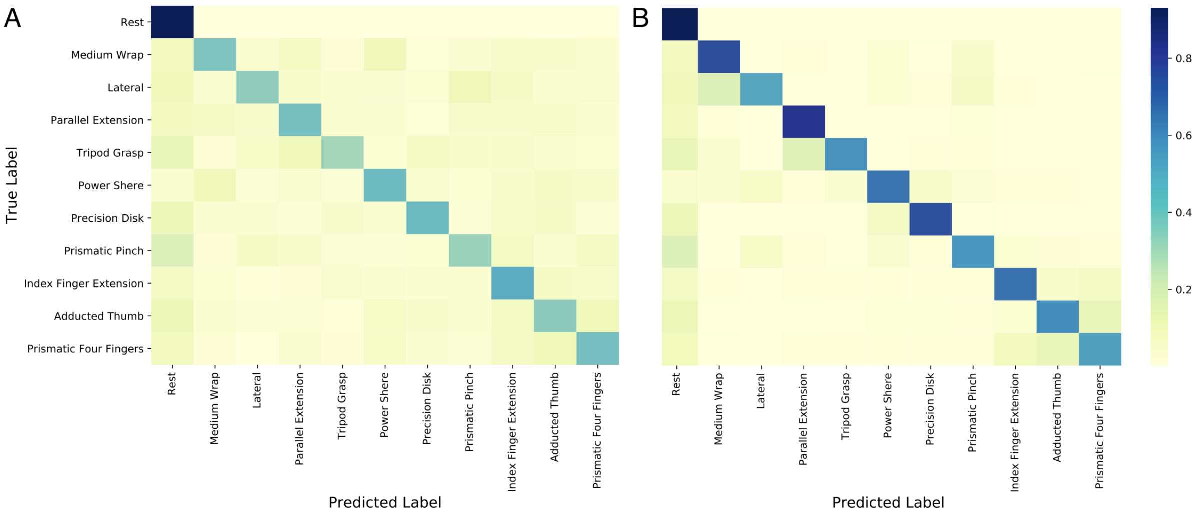

Figure S2. Normalized confusion matrices of sEMG-based and multimodal grasp-type classifications in transradial amputees for the dynamic condition. **(A)** Confusion matrix of sEMG-based grasp-type classification in transradial amputees. **(B)** Confusion matrix of multimodal grasp-type classification in transradial amputees.

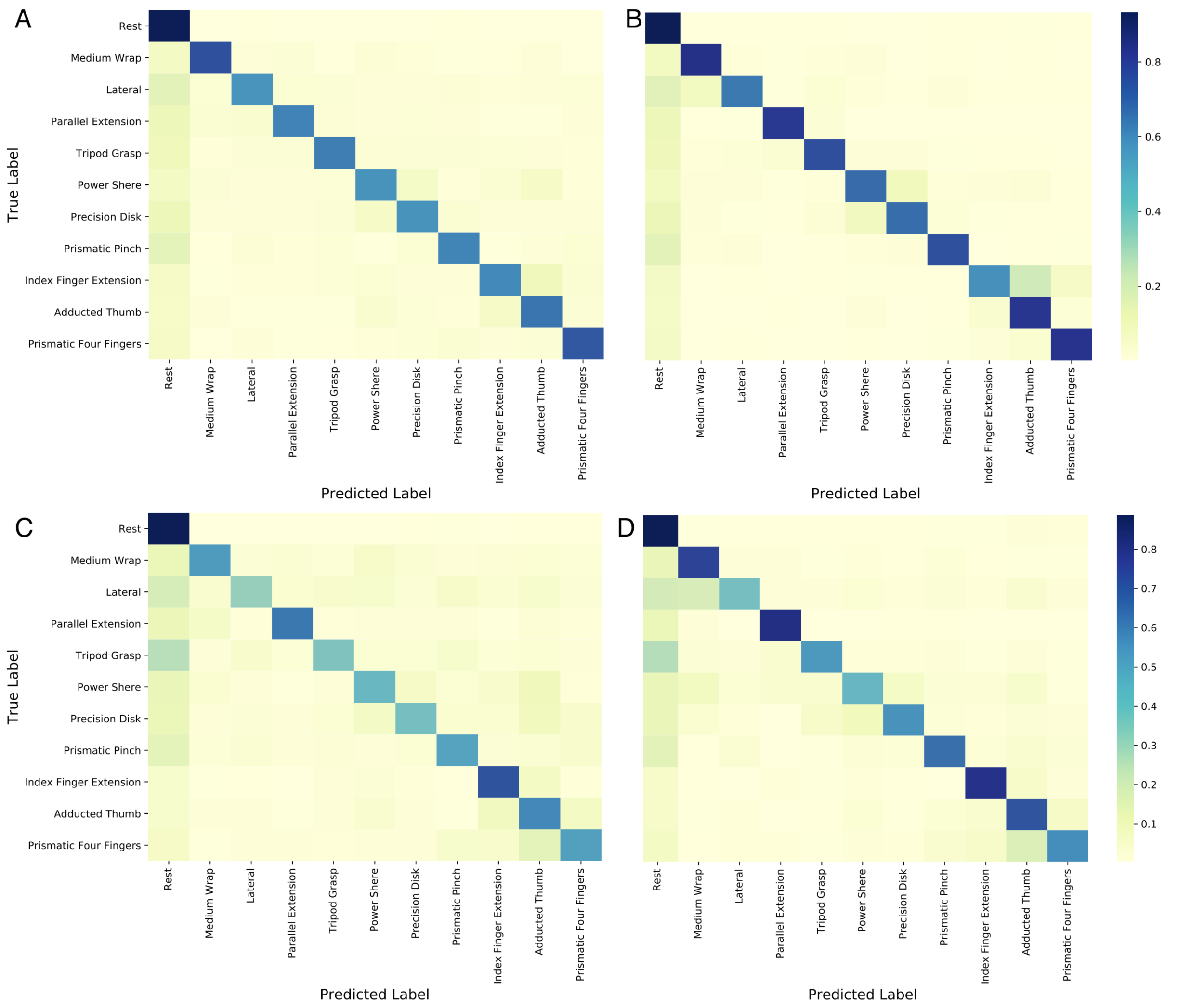

Figure S3. **(A)** Confusion matrix of sEMG-based grasp-type classification in able-bodied subjects for the static condition. **(B)** Confusion matrix of multimodal grasp-type classification in able-bodied subjects for the static condition. **(C)** Confusion matrix of sEMG-based grasp-type classification in able-bodied subjects for the dynamic condition. **(D)** Confusion matrix of multimodal grasp-type classification in able-bodied subjects for the dynamic condition.
